# Genetic profiling of different phenotypic subsets of breast cancer stem cells (BCSCs) in breast cancer patients

**DOI:** 10.1186/s12935-022-02841-2

**Published:** 2022-12-31

**Authors:** Abdel-Rahman N. Zekri, Abeer Bahnassy, Magda Mourad, Ibrahim Malash, Ola Ahmed, Mona S. Abdellateif

**Affiliations:** 1 Virology and Immunology Unit, Cancer Biology Department, National Cancer Institute, Cairo University, Cairo, Egypt; 2 Pathology Department, National Cancer Institute, Cairo University, Cairo, Egypt; 3 Medical Oncology Department, National Cancer Institute, Cairo University, Cairo, Egypt; 4 Medical Biochemistry and Molecular Biology, Cancer Biology Department, National Cancer Institute, Cairo University, Cairo, Egypt

**Keywords:** Stem cells, Breast cancer, CD44, CD24, EpCAM, EMT and *NOTCH*

## Abstract

**Background:**

Breast cancer stem cells (BCSCs) have a crucial role in breast carcinogenesis, development, and progression. The aim of the current study is to characterize the BCSCs through the genetic profiling of different BCSCs phenotypic subsets to determine their related genetic pathways.

**Methods:**

Fresh tumor tissue samples were obtained from 31 breast cancer (BC) patients for (1) Mammosphere culture. (2) Magnetic separation of the BCSCs subsets using CD24, CD44, and CD326 Microbeads. (3) Flow cytometry (FCM) assay using CD44, CD24, and EpCAM. (4) RT-PCR profiler Arrays using stem cell (SC) panel of 84 genes for four group of cells (1) CD44^+^/CD24^−^/EpCAM^−^ BCSCs, (2) CD44^+^/CD24^−^ /EpCAM^+^ BCSCs, (3) mammospheres, and (4) normal breast tissues.

**Results:**

The BCSCs (CD44^+^/CD24^−^/EpCAM^−^) showed significant downregulation in 13 genes and upregulation in 15, where the *CD44*, *GJB1* and *GDF3* showed the maximal expression (P = 0.001, P = 0.003 and P = 0.007); respectively).

The CD44^+^/CD24^−^/EpCAM^+^ BCSCs showed significant upregulation in 28 genes, where the *CD44, GDF3,* and *GJB1* showed maximal expression (P < 0.001, P = 0.001 and P = 0.003; respectively). The mammospheres showed significant downregulation in 9 genes and a significant upregulation in 35 genes. The maximal overexpression was observed in *GJB1* and *FGF2* (P = 0.001, P = 0.001; respectively).

The genes which achieved significant overexpression in all SC subsets were *CD44, COL9A1, FGF1, FGF2, GDF3, GJA1, GJB1, GJB2, HSPA9,* and* KRT15.* While significant downregulation in *BMP2, BMP3, EP300,* and *KAT8.*

The genes which were differentially expressed by the mammospheres compared to the other BCSC subsets were *CCND2, FGF3, CD4*, *WNT1*, *KAT2A, NUMB*, *ACAN, COL2A1*, *TUBB3*, *ASCL2, FOXA2, ISL1*, *DTX1,* and *DVL1.*

**Conclusion:**

BCSCs have specific molecular profiles that differ according to their phenotypes which could affect patients’ prognosis and outcome.

## Background

Breast cancer (BC) is a major health problem in females worldwide, as it represents the second leading cause of cancer-related deaths in women. It had been estimated that in 2020 there were 2.261 million new cases (11.7% of all sites) and 0.685 million deaths (6.9% of all sites) from BC globally [1]. Breast cancer is a heterogeneous disease with a multifactorial etiology. It is divided into distinct pathological subtypes including ductal, lobular, and mucinous carcinomas. Also, it has variable molecular characteristics according to estrogen receptor (ER), progesterone receptor (PR) expression, and HER2 amplification. In addition, it had been classified according to the transcriptome-based classifications into luminal and basal breast cancers [2–4].

Despite the variability of the treatment modalities and diagnostic tools available for BC patients, still there is an increased incidence of metastasis and adverse outcomes. Therefore, it is important to understand the underlying molecular mechanisms involved in the carcinogenesis and progression of BC [5].

Breast cancer stem cells (BCSCs) represent a subpopulation of tumor cells that possess the ability to self-renew, divide indefinitely, and differentiate into other types of cells according to the surrounding growth factors [6]. There is accumulating evidence proposed that BCSCs are the leading cause of cancer progression, metastasis, as well as resistance against antitumor chemo/ radio or hormonal therapy [7, 8]. The BCSCs are characterized by surface markers expression of CD44^+^/CD24^−/low^, as well as mammosphere formation [5]. The mammospheres can be developed by culturing in non-adherent non-differentiating culture conditions, which allow for the promotion of cells that are capable of survival and continuous proliferation in culture as discrete spherical clusters [9]. These mammosphere culture systems are used to identify and enrich putative BCSCs.

The CD44 is a non-kinase cell surface glycoprotein that binds to hyaluronic acid (HA) and mediates the interaction of the BCSCs and the surrounding matrix metalloprotease (MMP) and osteopontin (OPN) [10, 11]. Therefore, CD44 is important for the stemness properties of the cancer cells, as well as the regulation of cell proliferation, differentiation, and survival [12, 13].

CD24 is a glycosylphosphatidylinositol-linked cell surface glycoprotein, that inhibits chemokine receptor-4 (CXCR4), and regulates cell metastasis and proliferation [14, 15]. It is usually downregulated on the surface of BCSCs, however, its expression on BCSCs is associated with adverse outcomes in the luminal A and triple-negative BC (TNBC) subtypes [16]. The CD24 and CD326 (EpCAM) are the main surface marker expressed on the surface of the mammary stem cell (MaSCs). The MaSCs are normally present in the adult mammary gland, and they are responsible for the maintenance of the ductal architecture [17]. These cells were also identified as proliferative heterogeneous stem cells/progenitors in the luminal types of breast cancer [18]. While the CD24^−/low^ CD44^+^ BCSCs were more commonly enriched in the basal-like subtype and less frequent in the luminal types [19]. The BC is characterized by a high degree of intratumor heterogeneity, as a single tumor may contain BCSCs with different phenotypes according to the molecular forms of the tumor [20, 21]. Therefore, the characterization of the BCSCs should not be relay on CD44^+^CD24^−/low^ only [5]. Other markers can be used for the characterization of BCSCs including the expression of the surface markers e.g., ALDH1, Prominin-1 (CD133), and CD131, their ability to form spheroid culture, as well as the expression of different molecular markers involved in maintaining the self-renewal, differentiation and stemness properties of the BCSCs [22, 23]. These signaling pathways include Notch, Wnt/β-catenin, Hedgehog (Hh), TNF-α/NF-Kβ, transforming growth factor-β (TGF-β), receptor tyrosine kinase (RTK), and Janus kinase/signal transducer and activator of transcription (JAK-STAT) pathways [24, 25].

Therefore, the aim of the current study is to characterize the BCSCs by genetic profiling of different BCSCs phenotypic subsets, and determination of their related genetic pathways. This will allow us to accurately define the possible impact of BCSCs on the development and progression of the BC, as well as their contribution to patients' response to treatment, outcomes, and survival rates. Hence it will open a new avenue for potential targeted therapy in BC patients.

## Methods

This is a retrospective cohort study included 31 patients who were histo-pathologically confirmed for BC. The study was conducted at the National Cancer Institute (NCI), Cairo University during the period from January 2019 to May 2021. All patients were subjected to full history taking, full clinical examination, complete laboratory, and radiological assessment. The normal control samples were obtained from the females who underwent reduction mammoplasty at the NCI surgical unit.

### Sample collection

Fresh tumor samples were obtained from the operation theatre in a sterile, 50 ml plastic Falcon tube containing 10 ml of Dulbecco’s modified Eagle’s medium (DMEM). The samples were transferred immediately to the tissue culture lab for processing. A section of the tumor was sent to the Pathology department for routine histopathology and immunohistochemistry [Estrogen receptors (ER), progesterone receptors (PR), Herceptin-2 receptors (Her-2), and Ki-67] work to confirm the diagnosis.

### Isolation of breast cancer cells

The neoplastic tissues were washed several times in Hanks Balanced Salt Solution (HBSS; Invtrogen) and minced with sterile blades into very small pieces (0.2–0.5 mm each). The single-cell suspension was obtained by enzymatic digestion using collagenase (50–100 units/ml in HBSS; lnvitrogen) according to the studied protocol. The cells were incubated for 4–18 h at 37 ℃, and then filtered using a sterile stainless steel or nylon mesh. The cell suspension was washed several times by centrifugation in HBSS, and then the pellets were re-suspended in 500 µl–1 ml of Dulbecco's Modified Eagle Medium (DMEM). Using the haemocytometer, the cells were counted and divided into two parts, one part was used for mammosphere culture, and the other part was used for cellular characterization by FCM using CD44, CD24 and cytokeratin or EpCAM monoclonal antibodies.

#### Mammosphere culture

The mammosphere culture was performed according to the method of Dontu et al*.* [26] with modifications [27, 28]. Briefly, the single isolated breast cancer cells were suspended in ultra-low attachment plates at a density of 4 × 10^5^ viable cells/mL in primary culture and 1000 cells/mL in each passage. The cells were cultured in DMEM/Ham F-12 media (1:1) supplemented with insulin (5 mg/mL), hydrocortisone (0.5 mg/ml), and epidermal growth factor (20 ng/mL; all from Invitrogen ltd., Paisley, Scotland). The cells were then seeded into six-well plates (2.5 ml/per plate) or T25 tissue culture flasks (5 ml per flask). The non-adherent cells were fed weekly; measured using the gridded lens. The mammospheres were enzymatically dissociated every 7 days to 2 weeks by incubation in 0.5% trypsin–EDTA solution (Invitrogen) for about 5–10 min at 37 ℃, then dispersed by pipetting with a 23-gauge needle. During the mammosphere dissociation, a subset of cells from each passage was subjected to subsequent morphologic evaluation by microscopic examination. In addition to immunohistochemistry assessment using primary antibodies for pan CK, CD44, CD24, and CD133 (Abcam, UK).

#### Characterization of breast cancer cells by flow cytometry

A portion of the cell suspension was used for flow cytometric characterization of breast cancer stem cells using the Cell Quest program for the following conjugated antibodies: CD45-FITC (lymphocyte marker), CD24-PE (cancer stem cell & epithelial marker), CD44-FITC (cancer stem cell marker), pan cytokeratin or cytokeratin 19-PE (epithelial marker) or EpCAM-PE (epithelial marker) according to manufacturers' instructions (Becton & Dickinson, R&D, Milteny). Appropriate isotype controls were included in all cases to determine the areas of non-specific staining and unstained cells from each sample were also analyzed as a negative control. Accordingly, five subsets of cells were identified in each stained cases: CD44 + / CK^−^ or EpCAM^−^ cells, CD44 + / CK^+^ or EpCAM^+^ cells, CD44^−^/CK^+^ or EpCAM ^+^ cells, CD44^+^/CD24^−/low^ cells and CD24 + cells.

#### Separation of the breast cancer stem cells (BCSCs)

After the preparation of single-cell suspension, magnetic separation of the BCSCs subsets was done using the LS separation column (Miltenyi Biotec B.V. & Co. KG) according to the manufacturers' instructions. The cells were separated by magnetic selection after staining with CD24, CD44 and CD326 (EpCAM) Micro Beads labeled with monoclonal antibodies. Finally, different subsets of cells were collected by magnetic separation and stored for subsequent RNA extraction. Accordingly, the cells were divided into three groups including G1: CD44^+^/CD24^−^/EpCAM^−^ cells; G2: CD44^+^/CD24^−/^/EpCAM^+^ cells; and G3: mammospheres,

#### Gene profiling array

##### RNA extraction and quantitative real-time PCR (qRT-PCR)

RNA was extracted and purified from the different groups of cells after magnetic separation using RNeasy Midi Kit (Cat. No. 74104, Qiagen) according to manufacturers' instructions. The qRT-PCR was done using the RT2 profiler array (Cat. No. 330401, Qiagen). As for the stem cells (SCs) profiling assay, the SABiosciences RT2 qPCR Master Mixes (Cat. No. 330522, Qiagen) was used to obtain the most accurate results from the PCR Array.

The PCR was performed in the MaxPro3000 real time PCR (Startagen). Regarding the 96 well plate array the following reagents were mixed in a 5-ml tube at the recommended concentrations using a multi-channel pipette: 2X SABiosciences RT2 qPCR Master Mix, diluted First Strand cDNA Synthesis Reaction (using 500 ng RNA) and H_2_O. The amplification cycles were formed of an initiation step at 95 ℃ for 10 m, followed by 40 cycles at 95 ℃ for 15 s and, 55 ℃ for 90 s. The cycle threshold (Ct) for each well was determined and the ΔΔCt method was used for data analysis by the instrument's software. The Ct values of the control wells were determined including the Ct value of genomic DNA Control (GDC) and if it was greater than 35, the level of genomic DNA contamination was considered too low to affect gene expression profiling results. The studied genes were illustrated in Table 1.

**Table 1:** The selected genes and their functions

Function	Genes
Cell cycle regulators	*APC, AXIN1, CCNA2, CCND1, CCND2, CCNE1, CDK1, CDC42, EP300, FGF1, FGF2, FGF3, FGF4, MYC, NOTCH2, PARD6A, RBI*
Chromosome and chromatin modulators	*KAT2A, HDAC2, KAT8, KAT7, RB1, TERT*
Genes regulating symmetric/asymmetric cell division	*DHH, NOTCH1, NOTCH2, NUMB, PARD6A*
Self-renewal markers	*HSPA9, KAT8, KAT7, NEUROG2, SOX1, SOX2*
Cytokines and growth factors	*BMP1, BMP2, BMP3, CXCL12, FGF1, FGF2, FGF3, FGF4, GDF2, GDF3, IGF1, JAG1*
Genes regulating cell–cell communication	*DHH, DLL1, GJA1, GJBI, GJB2, JAG1*
Cell adhesion molecules	*APC, BGLAP, CD4, CD44, CDH1, CDH2, COL9A1, CTNNA1, CXCL12, NCAM1*
Metabolic markers	*ABCG2, ALDH1A1, ALDH2, FGFR1*
Stem cell differentiation markers	Embryonic Cell Lineage markers: *ACTC1, ASCL2, FOXA2, PDX1 (IPF1), ISL1, KRT15, MSX1, MYODI, T*
	Hematopoietic Cell Lineage Markers: *CD3D, CD4, CD8A, CD8B, MME*
	Mesenchymal Cell Lineage Markers: *ACAN (AGC1), ALP1, BGLAP, COL1A1, COL2A1, COL9A1, PPARG*
	Neural Cell Lineage Markers: *CD44, NCAM1, SIGMAR1, S100B, TUBB3*
Signaling pathways-portal or stem cell maintenance	Notch Pathway: *DLL1, DLL3, DTX1, DTX2, DVL1, EP300, KAT2A, HDAC2, JAG1, NOTCH1, NOTCH2, NUMB*
	Wnt Pathway: *ADAR, APC, AXIN1, BTRC, CCND1, FRAT1, FZD1, MYC, PPARD, WNT1*

### Data analysis

Data management and analysis were performed using statistical software package SPSS, version 22 (IBM, Armonk, Ny, USA). The flow cytometry data were presented as median and interquartile ranges (IQR) according to the performed normality test. Comparison between data were analyzed using Mann–Whitney. The PCR Array Data Analysis Web Portal presents the results in a tabular format, a scatter plot, a three-dimensional profile, and a volcano plot. All tests of hypotheses were performed at the alpha level of 0.05, with a 95% confidence interval.

## Results

### Patients’ characteristics

The median age of the recruited BC females was 47 (range; 22–68) years, and the mean age was 48.1 ± 11.4 years. There were 15 (48.4%) patients with grade 2 tumor, and 16 (51.6%) patients with grade 3. Lymph node (LN) involvement was encountered in 22 (71%) patients, and capsular invasion was detected in 18 (58.1%) BC patients. The ER and PR were expressed in the tumor tissue of 14 (45.2%) BC females, while HER2 was expressed in the tumor tissue of 7 (22.6%) patients. In addition, there were 8 (25.8%) patients positive for distant metastasis (Table 2).

**Table 2:** Clinico-pathological characteristics of the assessed breast cancer patients

		Frequency (N = 31)	Percent (%)
Age		48.1 ± 11.4	
		47 (22–68)	
Grade	2	15	48.4
	3	16	51.6
Tumor type	IDC	26	83.9
	ILC	3	9.7
	IDC + signet ring	1	3.2
	IDC + IBC	1	3.2
LN	Negative	9	29.0
	Positive	22	71.0
Distant metastasis	Negative	23	74.2
	Positive	8	25.8
ER	Negative	17	54.8
	Positive	14	45.2
PR	Negative	17	54.8
	Positive	14	45.2
Her2	Negative	24	77.4
	Positive	7	22.6
DCIS	Negative	22	71.0
	Positive	9	29.0
Capsular invasion	Negative	13	41.9
	Positive	18	58.1

### Mammosphere culture of the primary breast cancer cells

The mammospheres formed of cells that are capable of surviving and proliferating as discrete clusters in non-adherent, non-differentiating culture conditions. Such spheroids, which are enriched in progenitor cells capable of differentiating along multiple lineages. The size of the mammospheres depends upon the proliferation of the cells which varied according to the severity and the aggressiveness of the disease.

**Fig. 1:**
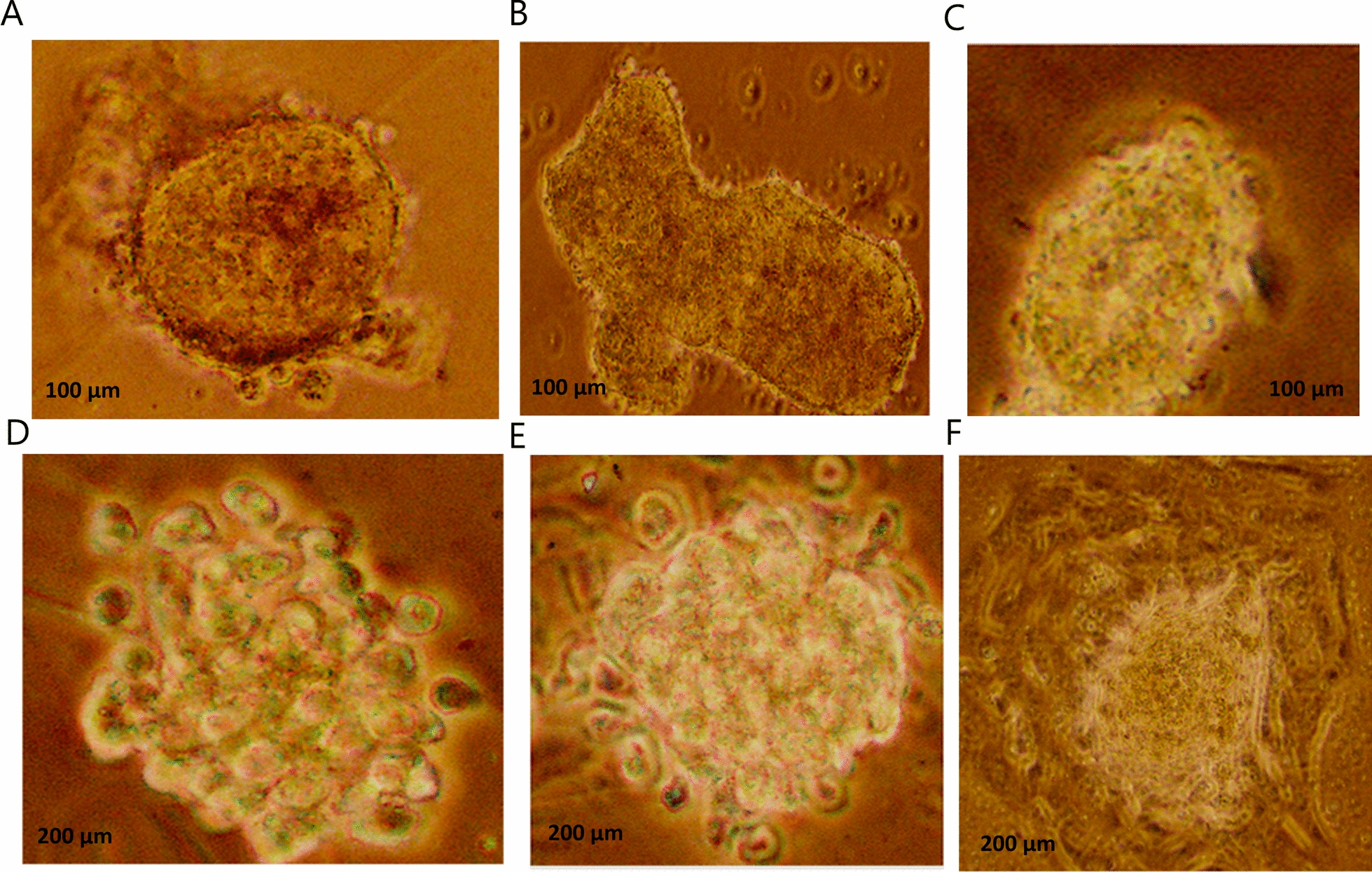
Discrete mammospheres of different sizes in non-differentiating conditions cultured from different cases. **A, B** and **C** showing mammosheres after one week (50–100 µm). **D, E** and **F**) showing mammosheres after three weeks (150–200 µm)

Viable mammospheres were produced in 13 out of the 31 cases included in the study (cultures were feasible for 15 cases only). In these cases, the mammospheres ranged in size from 20 to 180 µm and were successfully cultured past the third passage (Fig. 1). Immunohistochemistry using lineage markers were performed to cell blocks obtained from mammospheres after the second passage as well as after full differentiation.

### Characterization of breast cancer stem cells by flow cytometry

The CD44^+^/CD24 ^low/−^ population has been isolated from the primary breast tumors. These cells were found to be enriched with tumorigenic cells. Though, cells expressing CD44 have been mentioned as breast cancer stem cells; however, a portion of neoplastic breast epithelium is expressing CD44 too. Therefore, we used a panel of markers that enabled us to identify and enumerate CD44 + /CD24 ^low/−^ cells (tumorigenic stem cells), CD44 + /CK- or CD44 + /EpCAM- cells (stem cells), CD44 + /CK + or CD44 + /EpCAM + cells (neoplastic breast epithelium).

**Fig. 2:**
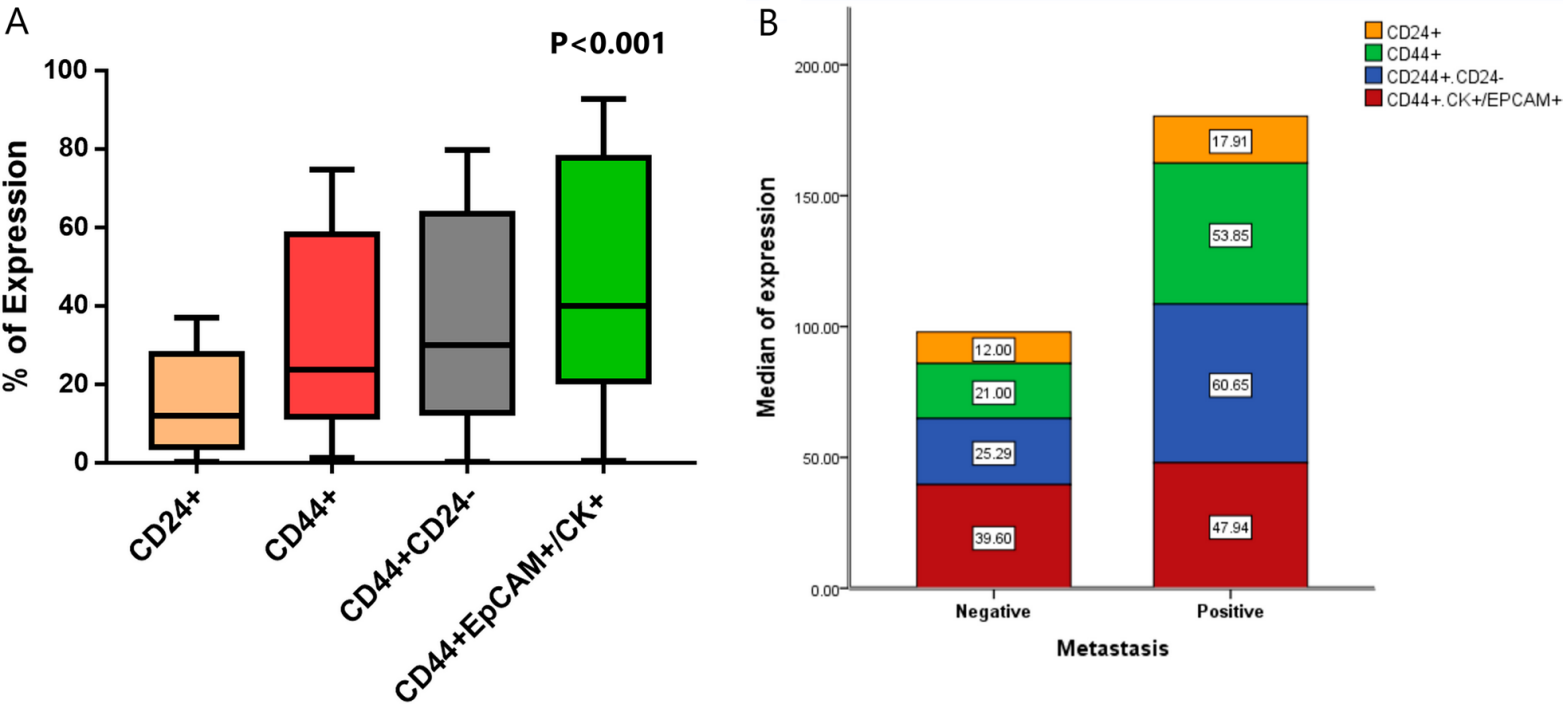
**A** Characterization of breast cancer stem cells by flow cytometry for the assessment of CD24 + , CD44 + , CD44^+^/CD24^−^, and CD44^+^ /EpCAM^+^/CK + cells in breast cancer patients. Cells expressing CD24 + was significantly down expressed compared to the other types of the assessed cells (P < 0.001 for all). There was a significant increase in Cells expressing CD44^+^/CD24^−^, and CD44^+^ /EpCAM^+^/CK + compared to those expressing CD24 + , and CD44 + . However, there was no significant difference between the number of cells expressing CD44^+^/CD24^−^, and those expressing CD44^+^ /EpCAM^+^/CK + (P = 0.123). **B** Association of metastasis and the expression markers of CD24 + , CD44 + , CD44^+^/CD24^−^, and CD44^+^ /EpCAM^+^/CK + cells in breast cancer patients

The neoplastic breast epithelium which expressed both CD44 and EpCAM or CD44 and CK was found to be 39.9% (range: 0.5–92.8%). Cells expressed CD44 only were 23.8% (range: 1.3–74.7%), and those expressed CD24 + only were 12% (range: 0.1–37%). While cells expressed CD44 + /CD24- were 29.9% (range: 0.1–79.7%, Fig. 2).

There was a significant association between CD44^+^/CD24 ^low/−^ expression and the ability of the tumor to metastasize, as the expression of CD44^+^/CD24 ^low/−^ in patients with distant metastasis was 61% (range: 7–80%), compared to 25.3% (range: 0.1–68%) in those who had not metastasize (P = 0.038). Notably, patients with increased CD44 + expression, showed increase incidence of metastasis, ER and PR expression, though it did not reach a significant level (P = 0.071, 0.059 and 0.059; respectively, Table 3).

**Table 3:** Association between the breast cancer cell subsets and patients’ clinical features

Clinical parameters		CD24 +	P value	CD44 +	P value	CD44 + CD24-	P value	CD44 + CK + /EPCAM +	P value
Grade	2	13.8 (0.1–37)	0.353	28 (3.6–66)	0.906	30 (0.1–68)	0.502	40 (6–88.7)	0.937
	3	11.7 (0.9–34)		22 (1.3–75)		34 (4.7–80)		41 (0.5–93)	
Tumor type	IDC	12.3 (0.9–37)	0.342	23.7 (1.3–75)	0.166	26 (2–80)	0.076	53 (0.5–93)	0.390
	ILC	12.6 (0.9–347)		59 (16–63)		63.5 (44.6–71)		40 (13–42)	
LN	− ve	11.5 (0.1–34)	0.273	21 (1–63)	0.507	22.5 (0.1–71)	0.654	65 (5.7–93)	0.219
	+ ve	12.5 (0.9–37)		26 (3.6–75)		33.5 (2–80)		36 (0.5–93)	
Metastasis	− ve	12 (0.1–37)	0.355	21 (1.3–66)	0.071	25.3 (0.1–68)	0.038	40 (0.5–93)	0.821
	+ ve	18 (4–34)		53.8 (6.5–75)		61 (7–80)		48 (3.5–79)	
ER	− ve	12 (0.1–34)	0.953	16.5 (1.3–66)	0.059	22.5 (0.1–71)	0.23	56 (3.5–93)	0.336
	+ ve	12 (0.9–37)		38.6 (3.6–75)		48 (2–79.7)		37.7 (0.5–87)	
PR	− ve	12 (0.1–34)	0.953	16.5 (1.3–66)	0.059	22.5 (0.1–71)	0.23	56 (3.5–93)	0.336
	+ ve	12 (0.9–37)		38.6 (3.6–75)		48 (2–79.7)		37.7 (0.5–87)	
HER2	− ve	12 (0.1–37)	1.00	28 (1.3–74.7)	0.473	33.5 (0.1–80)	0.695	41 (3.5–93)	0.872
	+ ve	12.5 (0.9–34)		23.7 (3.6–58)		30 (4–64.8)		40 (0.5–87)	
Capsular invasion	− ve	11.6 (0.1–43)	0.708	23.8 (1.3–66)	0.953	37 (0.1–71)	0.798	40 (5.7–93)	0.828
	+ ve	12.3 (0.9–37)		26 (3.6–74.7)		28 (2–79.7)		49 (0.5–93)	

### Data of the profiling array

**Fig. 3:**
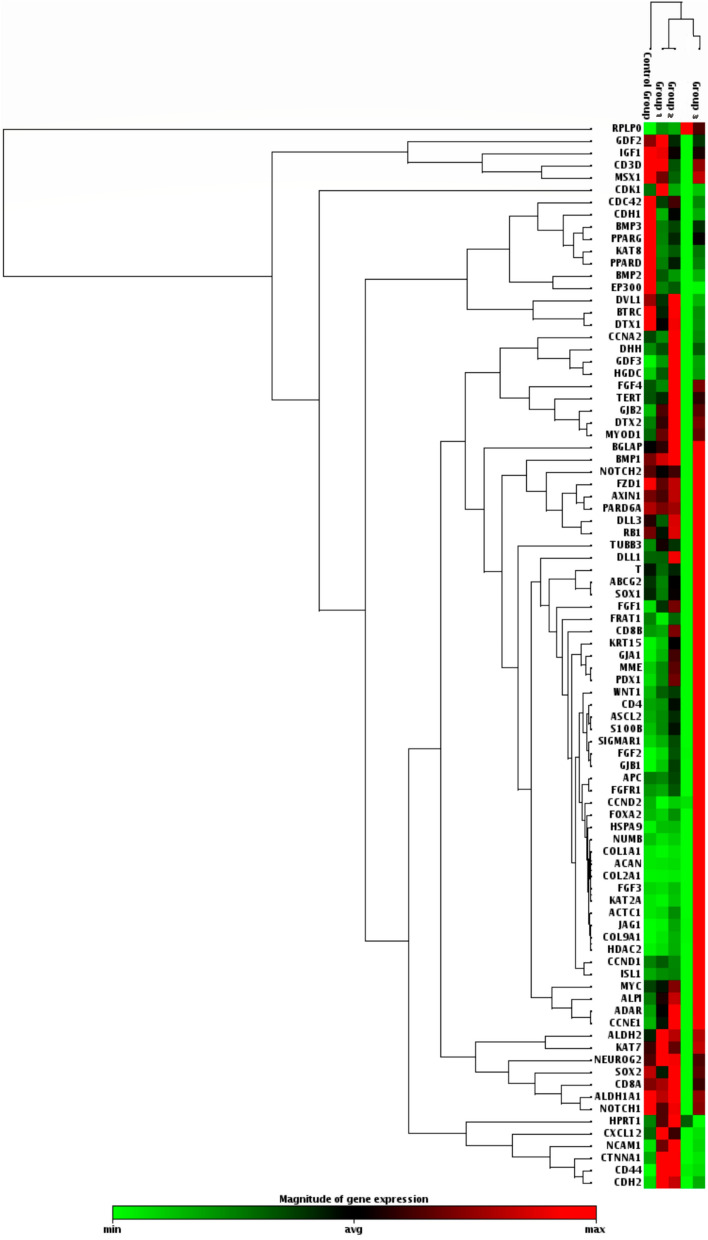
Unsupervised hierarchical clustering of the differentially expressed genes in breast cancer stem cells versus normal breast tissues

**Fig. 4:**
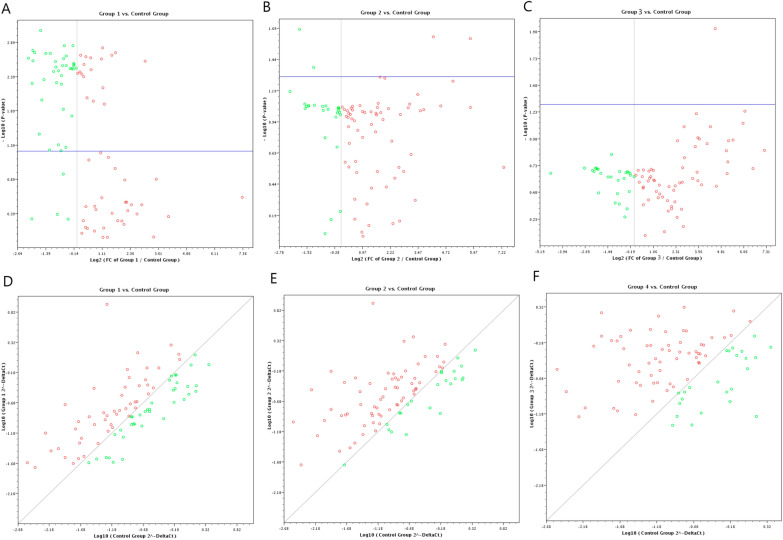
Scatter plot for the differentially expressed genes in CD44^+^/CD24^−^/EpCAM^−^ BCSCs **A, D**, CD44^+^/CD24^−^ /EpCAM^+^ BCSCs **B, E**, and mammospheres **C, F**, compared to normal breast tissue samples

The profiler Arrays was performed using SC panel of 84 genes which included SC specific markers, SC differentiation markers, and signaling pathway markers for SC (Fig. 3). The profiling genes were assessed in four groups including G1: BCSCs (CD44^+^/CD24 ^low/−^/EpCAM^−^), G2: BCSCs (CD44^+^/CD24^low/^ /EpCAM^+^), and G3: cultured mammospheres, compared to G4: the control normal group (Fig. 4).

Out of the 84 studied genes, the BCSCs (CD44^+^/CD24^−^/EpCAM^−^) showed that there were 13 significantly down-regulated genes which are involved in Cytokines and Growth Factors (*BMP2, BMP3, CCND2, CDC42, EP300*)*, Wnt* Pathway (*BTRC, FRAT1, PPARD*), Cell Adhesion Molecules (*CDH1*), Mesenchymal Cell Lineage Markers (*COL1A1, PPARG*), and Chromosome and Chromatin Modulators (*KAT2A, KAT8*). While there were 15 genes that significantly up-regulated which were involved in cell adhesion (*CD44, CDH2, COL9A1, CTNNA1, CXCL12, NCAM1*), cytokines and growth factors (*FGF1, FGF2, CXCL12, GDF3,*), cell–cell communication (*GJA1, GJB1, GJB2*), cell cycle regulation (*CDK1*), self-renewal (*HSPA9*), and embryonic Cell Lineage markers (*KRT15*). Where the *CD44* showed the maximal expression (fold regulation: 161.48, P = 0.001), followed by *GJB1* and *GDF3* (fold regulation: 16.56 (P = 0.003) and 11.37 (P = 0.007); respectively) compared to the control group.

Regarding the CD44^+^/CD24^−^/EpCAM^+^ BCSCs, the significantly downregulated genes were involved in cytokines and growth factors (*BMP2, BMP3, EP300*)*,* embryonic cell lineage markers (*MSX1),* chromosome and chromatin modulators (*KAT8*). While there were 28 genes that showed significant differential upregulation compared to the control group. These genes were involved in cell cycle regulation (*CCNA2, CCNE1, FGF2, FGF4*), cell adhesion molecules (*CD44, CDH1, CDH2, COL9A1, NCAM1*), cytokines and growth factors (*FGF1, FGF2, FGF4, GDF3*), cell–cell communication (*GJA1, GJB1, GJB2, JAG1, DHH*), self-renewal markers (*HSPA9*), *Wnt* pathway (*ADAR*), Notch pathway (*DTX2, HDAC2*, *JAG1*), mesenchymal cell lineage markers (*ALPI*), neural cell lineage markers (*NCAM1, S100B, CD44, SIGMAR1*), hematopoietic cell lineage markers (*CD8B, MME*), and embryonic cell lineage markers (*ACTC1, KRT15, MYOD1, PDX1*), Where the *CD44* showed the maximal expression (fold regulation: 158.5, P < 0.001), followed by *GDF3* and *GJB1* (fold regulation: 55.9 (P = 0.001) and 55.8 (P = 0.003); respectively) compared to the control group.

**Fig. 5:**
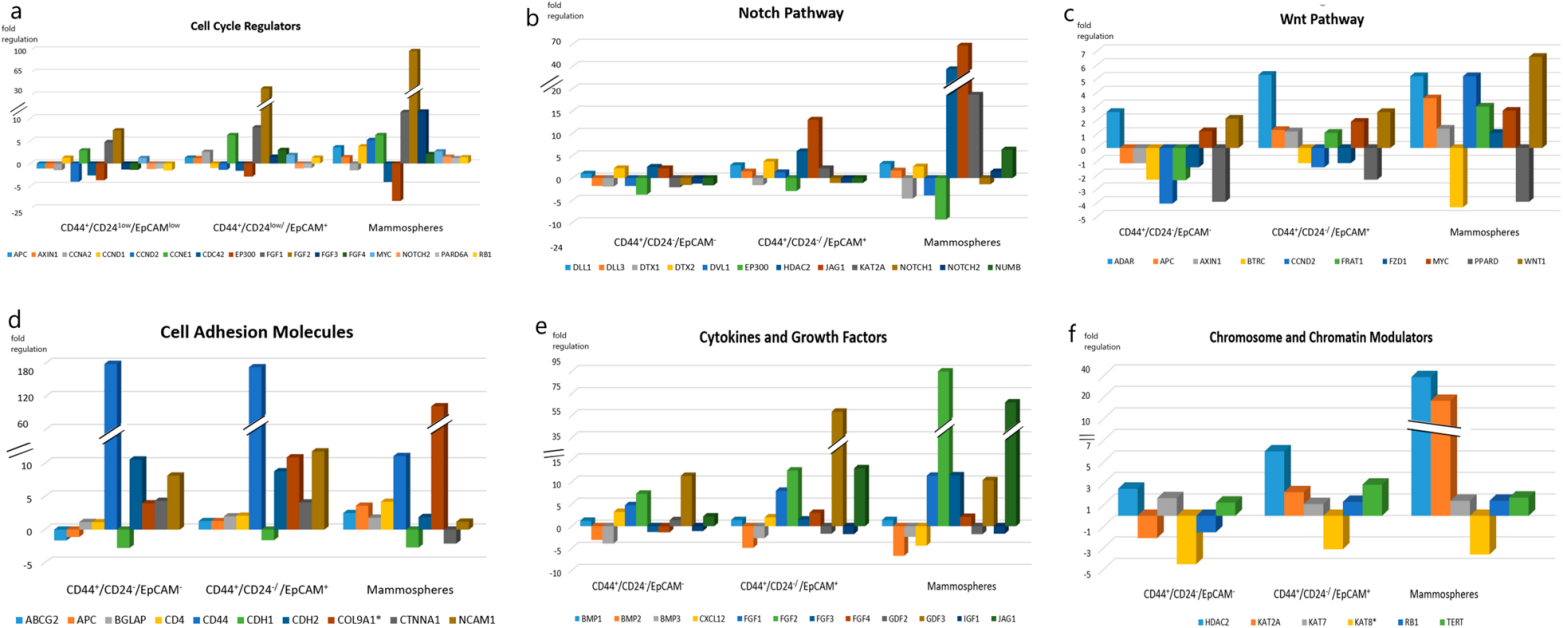
The expression profile of genes involved in **a** Cell cycle regulation, **b** Notch pathway, **c** Wnt pathway, **d** Cell adhesion molecules, **e** Cytokines and growth factors, **f** Chromosome and chromatin modulators, assessed in CD44^+^/CD24^−^/EpCAM^−^ BCSCs, CD44^+^/CD24^−^ /EpCAM^+^ BCSCs, and mammospheres

**Fig. 6:**
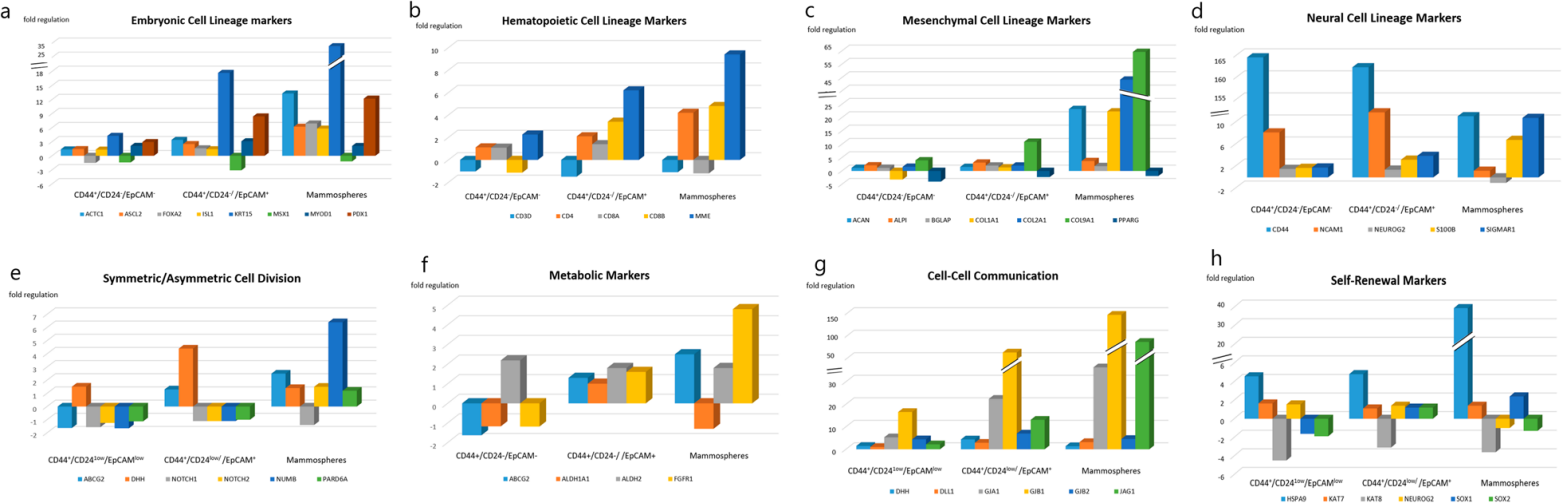
The expression profile of genes involved in **a** Embryonic cell lineage markers, **b** Hematopoietic cell lineage markers, **c** Mesenchymal cell lineage markers, **d** Neural cell lineage markers, **e** Symmetric/asymmetric cell division, **f** Metabolic markers, **g** Cell–cell communication, **h** Self-Renewal markers, assessed in CD44^+^/CD24^−^/EpCAM^−^ BCSCs, CD44^+^/CD24^−^ /EpCAM^+^ BCSCs, and mammospheres

Regarding the differential gene expression in the mammospheres, there were a significant downregulation in 9 genes which involved in cell cycle regulation (*CDC42*, *EP300*)*,* chromosome and chromatin modulators (*KAT8*), *Wnt* Pathway (*PPARD, BTRC*), cytokines and growth factors (*BMP2, CXCL12*), Notch pathway (*DTX1, DVL1*). While a significant upregulation in 35 genes which involved in cell cycle regulation (*CCND1, CCND2, CCNE1, CDC42, FGF1, FGF2, FGF3)*, cell adhesion molecules (*CD4, CD44, COL9A1*), cytokines and growth factors (*CXCL12, FGF1, FGF2, FGF3, GDF3, JAG1*), cell–cell communication (*GJA1, GJB1, GJB2, JAG1*), self-renewal markers (*HSPA9*), *Wnt* pathway (*ADAR, CCND1, WNT1*), Notch pathway (*HDAC2*, *JAG1, KAT2A, NUMB*), mesenchymal cell lineage markers (*ACAN, COL1A1, COL2A1, COL9A1*), neural cell lineage markers (*S100B, CD44, SIGMAR1, TUBB3*), hematopoietic cell lineage markers (*CD8B, MME*), embryonic cell lineage markers (*ACTC1, ASCL2, FOXA2, ISL1, KRT15, PDX1*), and a metabolic marker (*FGFR1)*. The maximal over expression was observed in *GJB1* (fold regulation: 147.5, P = 0.001), followed by* FGF2, JAG1* and* COL9A1* (fold regulation: 94.9 (P = 0.001), 69.7 (P = 0.001) and 64.6 (P = 0.040); respectively) compared to the control group (Table 4, Figs. 5, 6).

**Table 4:**
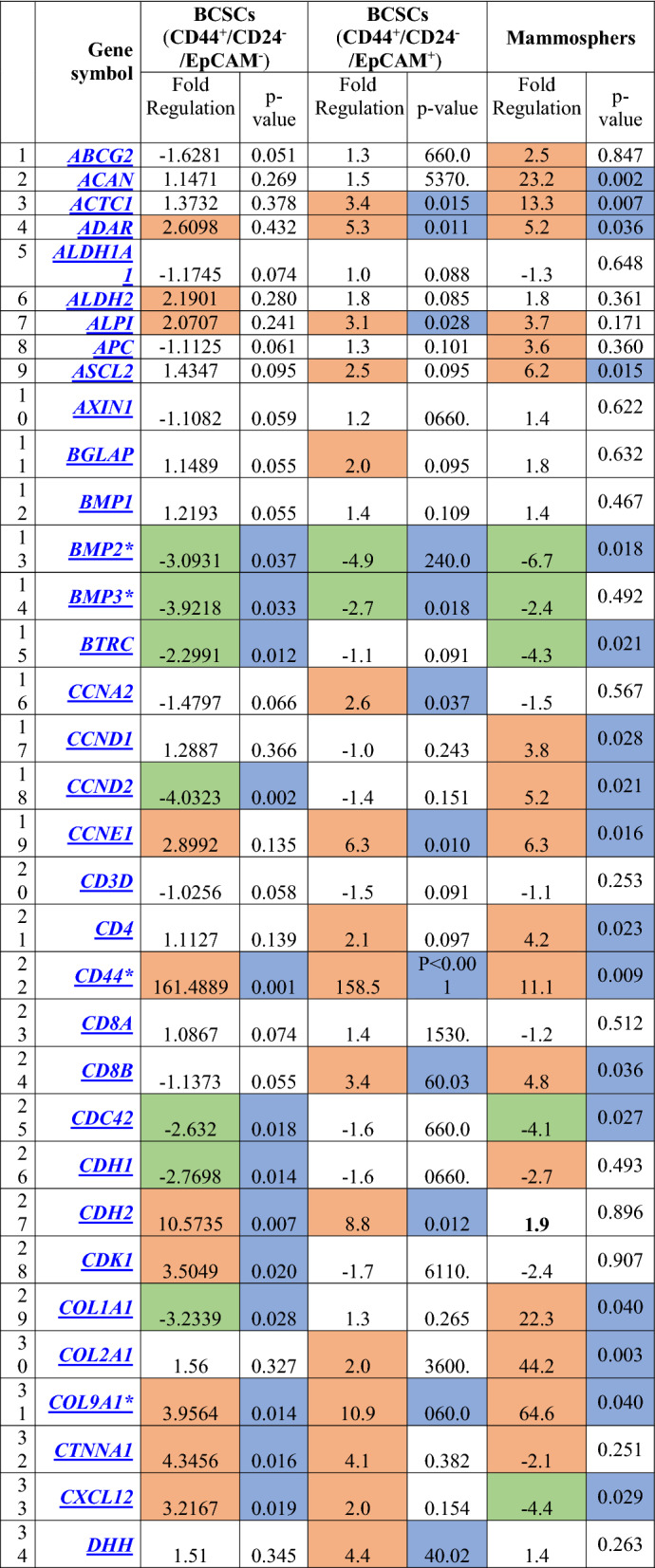

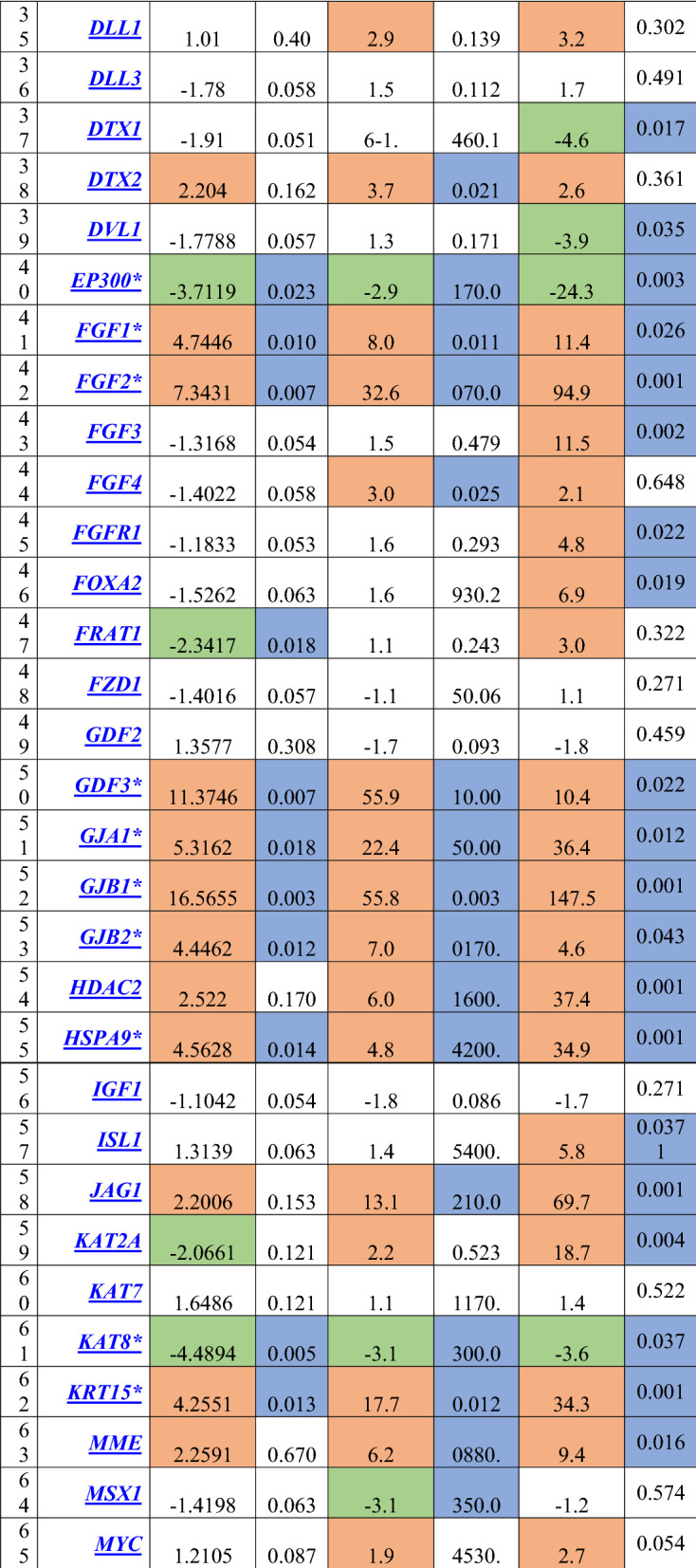

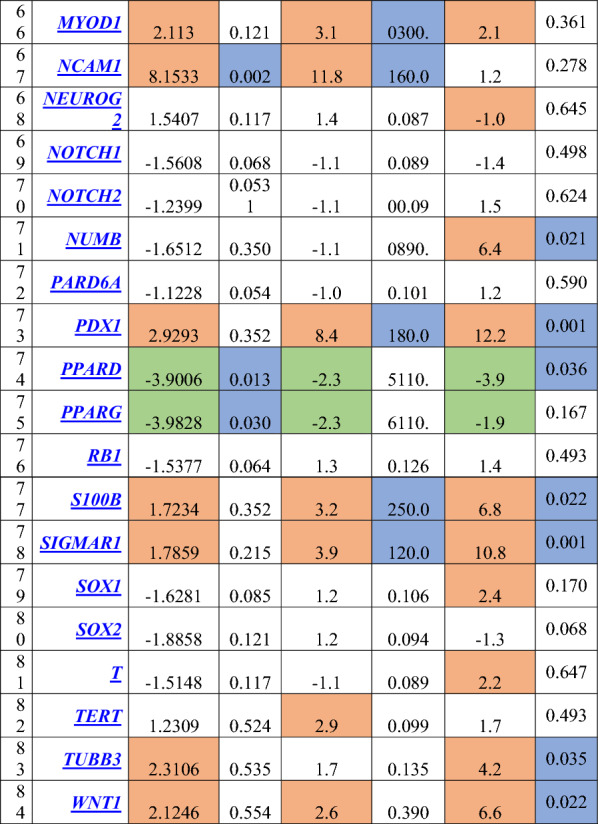
Gene profiling expression in BCSCs and Mammospheres in relation to the normal breast tissues

Cells in orange color denoted up-regulated genes, cells in green color denoted downregulated genes, cells in blue color denoted differentially significant expression, p-value is significant if <0.05.

*indicated the significantly expressed genes in the assessed three groups of cells.

The differentially overexpressed markers in all BCSCs subsets compared to the control group were those mostly involved in cell cycle regulation (*CCNE1, EP300, FGF1, FGF2*), cell adhesion molecules (*CD44, CDH2 COL9A1, CTNNA1*), cytokines and growth factors (*FGF1, FGF2, GDF3, JAG1*), cell–cell communication (*GJA1, GJB1, GJB2, JAG1*), self-renewal markers (*HSPA9*), *Wnt* pathway (*ADAR, WNT1*), Notch pathway (*DTX2, HDAC2*, *JAG1*), mesenchymal cell lineage markers (*ALPI, COL9A1*), neural cell lineage markers (*S100B, CD44, SIGMAR1*), hematopoietic cell lineage markers (*MME*), and embryonic cell lineage markers (*KRT15, MYOD1, PDX1*). While the most commonly down regulated genes in all studied groups compared to the control were *CDC42* (cell cycle regulation), *BMP2, BMP3,* (cytokines and growth factors), *KAT8* (chromosome and chromatin modulators), *PPARD, BTRC* (*Wnt* Pathway), *EP300* (*Notch* pathway)*,* and *PPARG* (mesenchymal cell lineage markers).

However, the genes which achieved significant over expression in all studied SC subsets were *CD44, COL9A1, FGF1, FGF2, GDF3, GJA1, GJB1, GJB2, HSPA9,* and* KRT15.* While those which significantly downregulated were *BMP2, BMP3, EP300,* and *KAT8.*

## Discussion

Breast cancer is a heterogenous disease characterized by variable genetical and phenotypical subtypes, which accordingly leads to diverse outcomes in BC patients. This heterogeneity of the BC cells is mainly linked to the cell of origin [29]. An increasing body of evidence reported that BC could be developed from dysregulation of the mammary stem cells. In the current study we tried to investigate the underlying molecular pathways involved in BC development and progression. We assessed three different subsets of BCSCs included (1) BCSCs (CD44 + /CD24 ^low/−^/EpCAM^−^), (2) BCSCs (CD44^+^/CD24^low/^ /EpCAM^+^), and (3) cultured mammospheres, through molecular profiling of 84 genes involved in the stemness properties of BCSCs, all were compared to normal breast tissue control.

The present data showed that the three subtypes of BCSCs shared over expression of common genes responsible for the self-renewal (*HSPA9*), cell cycle regulation (*CCNE1, EP300, FGF1, FGF2*), cell adhesion (*CD44, CDH2 COL9A1, CTNNA1*), cell–cell communication (*GJA1, GJB1, GJB2, JAG1*), expression of some cytokines and growth factors (*FGF1, FGF2, GDF3, JAG1*), and expression of differentiating cell lineage including mesenchymal markers (*ALPI, COL9A1*), neural markers (*S100B, CD44, SIGMAR1*), hematopoietic markers (*MME*), and embryonic cell lineage markers (*KRT15, MYOD1, PDX1*). In addition, they express some genes required for the stemness properties of the recruited cells included those involved in *Wnt* pathway (*ADAR, WNT1*) and Notch pathway (*DTX2, HDAC2*, *JAG1*). However, each type of cells has its own characteristically differential gene expression according to its phenotype, which later will formulate the tumor behavior and patients’ outcomes. In consistent with these data, many published series illustrated the importance of Wnt pathway, hedgehog pathway, and notch pathway for maintain the tumorigenicity, self-renewal and epithelial-mesenchymal transition (EMT) properties of the cancer stem cells [30–32].

The current study demonstrated that the CD44^+^/CD24^−^/EpCAM^+^ BCSCs, showed significant differential upregulation in some genes when compared to the CD44^+^/CD24^−^/EpCAM^−^ BCSCs.

These genes were those involved in cell cycle regulation (*CCNA2, CCNE1, FGF2, FGF4*), cell adhesion molecules (*CDH1, NCAM1*), cytokines and growth factors (*FGF4,*), cell–cell communication (*JAG1, DHH*), *Wnt* pathway (*ADAR*), *Notch* pathway (*DTX2, HDAC2*, *JAG1*), mesenchymal cell lineage markers (*ALPI*), neural cell lineage markers (*NCAM1, S100B, CD44, SIGMAR1*), hematopoietic cell lineage markers (*CD8B, MME*), and embryonic cell lineage markers (*ACTC1, MYOD1, PDX1*). In agreement with these data, Wu et al. [33], found that the gene expression signature associated with EMT in BCSCs were *ITGA6, EPCAM, CCND1, CD44, EGFR, CDH1*, and *MKI67*. Luo et al. [6], reported also that the CD44^+^/CD24^−^/EpCAM^+^ BCSCs play an important role in tumor metastasis, as it showed increased expression of genes responsible for EMT. In addition to increased expression of inflammatory cytokines and proteins associated with tumor invasion and metastasis.

Our data showed also that CD44^+^/CD24^−^/EpCAM^+^ BCSCs are more aggressive and tumorgenic than the CD44^+^/CD24^−^/EpCAM^−^ BCSCs denoted by the differential expression of genes involved in *Wnt* and *Notch* pathway, as well as the increased expression of mesenchymal, embryonic, and neural cell lineage markers. These findings are consistent with Luo et al. [6], who reported that the BCSCs CD44^+^/CD24^−^ positive for EpCAM showed increased incidence of treatment resistance and tumor recurrence. Similarly, Al-Hajj et al. [34], concluded that the BCSC (CD44^+^/CD24^−^) which express EpCAM on their cell surface, were more tumorigenic and had potent invasive properties when transplanted in immunodeficient mice in comparison to those lacking the expression of EpCAM.

Thus, the increased expression of EpCAM on the surface of BCSCs confers an aggressiveness, metastasis, drug resistance, and tumorigenic properties for the BC, which consequently associated with poorer outcomes in the BC patients.

Furthermore, both groups of BCSC subsets (CD44^+^/CD24^−^/EpCAM^+^ and CD44^+^/CD24^−^/EpCAM-) showed that the maximal expression was observed in CD44, followed by GDF3 (Growth differentiation factor-3) and GJB1 (Gap junction beta-1 protein). These data are in line with many series reported that CD44 has a fundamental role in EMT as it acts as an adhesion molecule and receptor for extracellular glycosaminoglycan hyaluronic acid, which leads to increased cell motility, and tumorigenicity [6, 35, 36]. This finding was confirmed by our results showing that CD44 expression was significantly increased in BC patients with distant metastasis.

Regarding the analysis of the gene expression profile of the cultured mammosphers, the current data demonstrated that the genes which were differentially overexpressed by the mammosheres when compared to the other BCSC subsets (CD44^+^/CD24^−^/EpCAM^+^ and CD44^+^/CD24^−^/EpCAM-), were those involved in cell cycle regulation (*CCND2, FGF3)*, cell adhesion molecules (*CD4*), *Wnt* pathway (*WNT1*), Notch pathway (*KAT2A, NUMB*), mesenchymal cell lineage markers (*ACAN, COL2A1*), neural cell lineage markers (*TUBB3*), and embryonic cell lineage markers (*ASCL2, FOXA2, ISL1*). While there was a differential downregulation in *DTX1, DVL1* which were involved in the Notch pathway. Theses exclusively upregulated genes in mammosphers confirmed the nature of the mammospher derived cells which allow for the selection of highly undifferentiated and aggressive cells with marked stemness properties. In line with these data, Dontu and his colleagues performed microarray analysis of mammosphers and other differentiated BC cells. They observed a significant upregulation of genes involved in growth hormone receptors, thrombin receptors, and Notch signaling pathway [26]. Another study done by Ramalho-Santos et al. also concluded the upregulation of Jak/Stat signaling, Notch signaling, increased transporter activity, DNA repair genes, growth hormone and thrombin receptors in mammospher derived cells [37].

Moreover, our data revealed that the maximal overexpressed genes in the mammosphers were the *GJB1* followed by* FGF2, JAG1* and* COL9A1* compared to the control group. These results are consistent with many previously published studies proposed that *GJB1* and *JAG1* associated significantly with the development of distant metastasis [38], while *COL9A1* and* FGF2* mutation associated with drug resistance in breast cancer patients [39, 40].

By comparing all studied groups, we identified 10 candidate genes which were significantly overexpressed in all BCSC subsets. These genes were important for cell cycle regulation (*FGF1, FGF2),* cell adhesion molecules (*CD44, COL9A1*), cytokines and growth factors (*FGF1, FGF2, GDF3*), cell–cell communication (*GJA1, GJB1, GJB2*), self-renewal markers (*HSPA9*), mesenchymal cell lineage markers (*COL9A1*), and embryonic cell lineage markers (*KRT15*). While those achieved significant downregulation were *BMP2, BMP3,* (cytokines and growth factors), *KAT8* (chromosome and chromatin modulators), and *EP300* (Notch pathway). It had been reported that KAT8 (K-lysine acetyltransferase 8), BMP2, BMP3 (Bone morphogenetic protein), and *EP300* were significantly downregulated in breast cancer, as they have a major role in controlling tumor progression through histone acetylation, metastasis suppression and inhibition of EMT, respectively [39–41].

## Conclusions

The current study provided evidence that BCSCs have specific molecular profiles that differ according to their phenotypes, which could affect patients’ prognosis and outcome. CD44 is an important marker for characterization of BCSCs, and its co-expression with EpCAM provides an aggressive and metastatic properties for the BCSCs rather than those with CD44^+^/CD24^−^/EpCAM^−^ phenotype. The mammosheres are the most aggressive type of breast cancer cells which exclusively had its own molecular profile including *CCND2, FGF3*, *CD4*, *WNT1*, *KAT2A, NUMB*, *ACAN, COL2A1*, *TUBB3 ASCL2, FOXA2, ISL1*, *DTX1,* and *DVL.* These genes could be considered as molecular markers for aggressiveness, metastasis, and resistance to treatment. Additionally, our data provided a panel of 14 genes (*CD44, COL9A1, FGF1, FGF2, GDF3, GJA1, GJB1, GJB2, HSPA9, KRT15, BMP2, BMP3, EP300,* and *KAT8*) which were expressed (up or downregulated) in all the assessed subsets of BCSCs which could serve as molecular markers with a potential diagnostic, prognostic and/or predictive value for breast cancer patients. However, the data of the profiling array should be validated on a larger number of patients, and also it should be correlated to the patients’ clinical courses in the form of distant metastasis, response to treatment and survival rates.

## Data Availability

All data generated or analyzed during this study are included in this published article.
